# Draft Whole-Genome Sequence of *Serratia marcescens* Strain MCB, Associated with *Oscheius* sp. MCB (Nematoda: Rhabditidae) Isolated from South Africa

**DOI:** 10.1128/genomeA.00911-14

**Published:** 2014-09-18

**Authors:** Mahloro H. Serepa, Vincent M. Gray

**Affiliations:** School of Molecular and Cell Biology, University of the Witwatersrand, Johannesburg, South Africa

## Abstract

Here we report on the draft genome sequence of *Serratia marcescens* strain MCB associated with *Oscheius* sp. MCB (Nematoda: Rhabditidae) isolated from South African soil. *S. marcescens* strain MCB has 5,304,212-bp genome size with 4,877 genes and a G+C content of 59.1%.

## GENOME ANNOUNCEMENT

*Serratia* is a genus of bacteria that is Gram negative, motile, rod-shaped, and belongs to the *Enterobacteriaceae* family. *Serratia* species have been isolated from plants, vertebrates, and invertebrates with over 70 species found to be associated with insects (1, 2). Recently *Serratia* bacteria were found to be mutually associated with the newly discovered entomopathogenic nematodes (EPNs) genera, *Oscheius carolinensis*, *O. chongmingensis*, and *O. rugaoensis* (3–6). The mutual relationship of this third group of EPNs, *Oscheius* with *Serratia* species has many similarities with the association that steinernematids and heterorhabditis have with the symbiotic bacteria *Xenorhabdus* and *Photorhabdus*, respectively. *Serratia* species associated with *Oscheius* EPN species also secretes various metabolites some of which have the ability to kill insect hosts and inhibit growth of competing bacterial and fungal species, as is the case with *Xenorhabdus* and *Photorhabdus* (7). One metabolite with antibiotic activity secreted by *Serratia marcescens* is prodigiosin (8, 9). Here we present the description of the draft genome sequence and the annotation of *S. marcescens* strain MCB associated with *Oscheius* sp. MCB (GenBank accession no. KF684370) (Nematoda: Rhabditidae) which was isolated from South Africa.

*S. marcescens* strain MCB was isolated from *Oscheius* sp*.* MCB nematodes according to methods described by Kaya and Stock (10). Genomic DNA was isolated from solid bacterial colony cultures using the ZR fungal/bacterial DNA MiniPrep kit (Zymo Research, catalog #D3050). The DNA extracted from the bacterial colonies was quantified using the NanoDrop ND-1000 spectrophotometer (Bio-Rad) and then cleaned with ZR fungal\bacterial DNA clean and concentrator-5 (catalog #D4003S). Genomic DNA paired-end libraries were generated with a NextEra DNA sample kit (Illumina) and indexed using a NextEra DNA index kit (Illumina). Paired-end (2×300 bp) sequencing was performed on MiSeq (Illumina) using the MiSeq reagent kit v3 at the Agricultural Research Council (ARC) Biotechnology Platform. Quality adapter trimming was performed on CLC Genomics Workbench v7 (CLC bio).

A total of 2,169,542 paired-end reads at 197× coverage were obtained from this workflow. The genome was assembled using the *de novo* assembly tool in the CLC bio, which produced 104 contigs with an average length of 51,002 bp and an *N*_50_ of 157,248 bp. The genome of *S. marcescens* strain MCB has 5,304,212 bp, with G+C (59.1%) content, which is similar to other *Serratia* species (2,11). Genome annotation was performed using the NCBI Prokaryotic Genome Automatic Annotation Pipeline (PGAAP). *S. marcescens* strain MCB genome has 4,877 genes, among the identified genes 4,756 are protein coding sequence genes (CDSs) and 21 are pseudogenes. The genome also has 14 rRNA genes with five operons (5S, 16S, and 23S) and 76 tRNAs genes. We found ten genes responsible for antibiotic synthesis and five virulence factors.

### Nucleotide sequence accession numbers.

This whole-genome shotgun project has been deposited at DDBJ/EMBL/GenBank under the accession JPQY00000000. The version described in this paper is version JPQY01000000.
